# Complete genome of *Penicillium herquei* HGN12.1C isolated from *Dysosma difformis* in Vietnam

**DOI:** 10.3389/ffunb.2026.1695025

**Published:** 2026-02-02

**Authors:** Duong Huy Nguyen, Nhung Thi Doan, Ngoc Bich Pham

**Affiliations:** 1 Institute of Biology (IB), Vietnam Academy of Science and Technology (VAST), Hanoi, Vietnam; 2 Graduate University of Science and Technology (GUST), Vietnam Academy of Science and Technology (VAST), Hanoi, Vietnam

**Keywords:** *Penicillium herquei*, *Dysosma difformis*, endophytic fungus, genome assembly and annotation, podophyllotoxin

## Introduction

*Penicillium herquei* HGN12.1C is an endophytic fungus isolated from *Dysosma difformis*, a native medicinal plant found in the mountainous provinces of northern Vietnam. Previous studies have demonstrated that *Penicillium herquei* HGN12.1C (Nguyen et al., 2024), along with several other endophytic fungi such as *Trametes, Purpureocillium, Aspergillus*, and *Ganoderma* (Tran et al., 2022), as well as the strain *Fusarium proliferatum* TQN5T (Nguyen et al., 2023), are capable of synthesizing podophyllotoxin (PTOX) and its derivatives.

PTOX is an important natural compound with numerous medical applications, including effective antimicrobial, antifungal, and anticancer properties (Ardalani et al., 2017; Yu et al., 2017). PTOX has been found in various plant species such as *Podophyllum hexandrum* (Anand et al., 2022), *Callitris intratropica* (Wanner et al., 2015), and *Juniperus horizontalis* Moench (Cantrell et al., 2014). However, studies on PTOX in fungi remain largely unexplored, particularly due to the lack of high-quality genome sequence data, which would provide valuable resources for comparative taxonomy as well as for investigating the biosynthetic pathways and mechanisms of bioactive compounds such as PTOX. Therefore, sequencing and exploring the genome of the fungal strain *Penicillium herquei* HGN12.1C is considered a crucial step for genomic analysis, comparative studies, and the identification of genes involved in the biosynthesis of PTOX and other bioactive compounds.

Here, we present the complete genome dataset of *Penicillium herquei* HGN12.1C to provide a valuable resource for comparative genomics and investigations of biosynthetic pathways.

## Value of data

Previous studies have characterized the biochemical features and biosynthetic pathways of podophyllotoxin in plants. In this work, we provide a high-quality genome of an endophytic fungus capable of biosynthesizing PTOX. The genome has been assembled and annotated with high-quality, enabling further exploration of taxonomic, genetic, and metabolic characteristics, as well as genes related to the biosynthetic pathways of secondary metabolites, especially PTOX, in fungi.

## Dataset description

**Figure 1:**
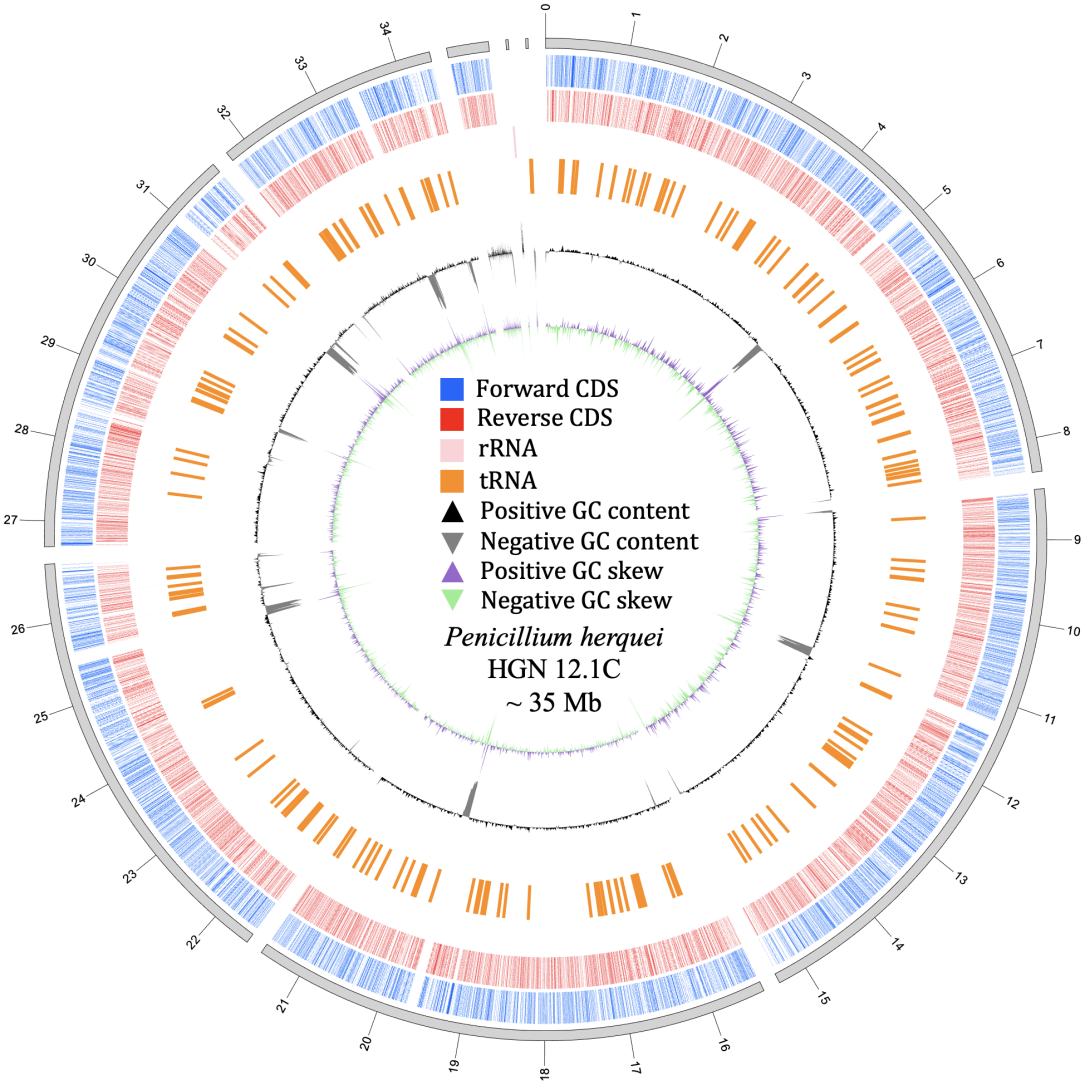
Structural overview of the *Penicillium herquei* HGN12.1C genome assembly. The eight nuclear chromosome-level contigs and one mitochondrial scaffold are concatenated for visualization purposes. The outermost ring indicates the genomic position scale in megabases (Mb). Concentric data tracks from the outside inward represent: (i) protein-coding genes (CDSs) on the forward strand (blue) and reverse strand (red); (ii) ribosomal RNA (rRNA) genes (pink); (iii) transfer RNA (tRNA) genes (orange); (iv) GC content deviation from the mean (black: positive; gray: negative); and (v) GC skew (purple: positive; green: negative).

The complete genome consists of nine contigs with a total length of 34,960,691 bp, an average read depth of 195×, and a GC content of 46.38% (**Figure 1**). It has an N50 value of 6,172,464 bp, an L50 of three, and the largest contig measures 8,398,835 bp. Among these, one contig (62,425 bp) was identified as the mitochondrial genome (**Supplementary Table S1**, **Supplementary Figure S1**). The nuclear genome comprises eight contigs, consisting of seven chromosome-level assemblies and one smaller unplaced scaffold (**Table 1**).

**Table 1:** Summary of genomic features and assembly statistics for strain HGN12.1C.

Genome assembly features		Values	
Total assembly size (bp)		34,960,691	
Total assembly size (≥ 1,000bp)		34,960,691	
Total assembly size (≥ 50,000bp)		34,903,660	
Number of scaffolds (≥1,000bp)		8*	
Number of scaffolds (≥50,000bp)		7	
Number of organelles		1	
Largest scaffold (bp)		8,398,835	
Genome size (bp)		34,960,691	
Mitochondrial size (bp)		29,635	
GC-content (%)		46.38	
N50 length chromosome (bp)		6,172,464	
L50 chromosome count		3	
Genome coverage		195×	
Completeness evaluation (%)			
Completeness		98.4	
Complete and single-copy BUSCOs		97.9	
Complete and duplicated BUSCOs		0.5	
Fragmented BUSCOs		0.8	
Missing BUSCOs		0.8	
Repeat annotation			
Simple repeat (bp)		4,164	
DNA transposon (bp)		16,386	
Total percentage of repeat sequences		0.06	
Total repeat length (bp)		20,550	
Genomic annotation			
*Predictors*	AUGUSTUS	SNAP	GlimmerHMM
Predicted raw genes	10,716	8,830	11,689
Predicted genes (after EVM)		11,054	
Protein coding genes		11,054	
tRNA		249	
rRNA (5S, 5.8S, 18S, 28S)		19 (3, 5, 7, 4)	
Annotated genes (total)		11,322	

*The assembly consists of seven chromosome-level contigs and one unplaced scaffold.

The dataset includes BUSCO assessment results showing 98.4% completeness (1,291 of 1,312 core genes detected). Repetitive sequences identified using standard fungal libraries accounted for 20,550 bp, representing 0.06% of the total assembled genome size. It should be noted that this value likely underestimates the total repetitive content, as *de novo* repeat identification was not performed.

The dataset includes annotation files generated by the Funannotate pipeline integrating AUGUSTUS, GlimmerHMM, and SNAP predictors, with final gene counts summarized in **Table 1**. After removing overlapping and redundant genes, 11,054 protein-coding genes were identified. Combined with 249 tRNA genes and 19 rRNA genes, the *Penicillium herquei* HGN12.1C genome was annotated with a total of 11,322 genes (**Table 1**).

The complete mitochondrial genome of *Penicillium herquei* strain HGN12.1C consists of a circular DNA molecule of 29,635 bp in length (**Supplementary Figure S1**). The mitogenome encodes 44 genes, comprising 15 protein-coding genes (PCGs), two ribosomal RNA (rRNA) genes, and 27 transfer RNA (tRNA) genes (**Supplementary Table S1**). The overall base composition is 36.9% A, 38.0% T, 11.6% C, and 13.4% G, resulting in a low GC content of 25.0%, which is consistent with other *Penicillium* species.

The 15 PCGs include seven NADH dehydrogenase subunits (*nad1–6, nad4L*), one cytochrome *c* reductase subunit (*cob*), three cytochrome *c* oxidase subunits (*cox1–3*), three ATP synthase subunits (*atp6, atp8, atp9*), and one ribosomal protein (*rps5*). The total length of these PCGs is approximately 16,500 bp, accounting for 55.7% of the entire mitogenome. The two rRNA genes include the small subunit (*rns*) and the large subunit (*rnl*). Notably, the *rnl* gene is fragmented into exons by the insertion of the *rps5* gene and introns. The set of 27 tRNA genes ranges in length from 71 to 86 bp and is sufficient to decode all 20 standard amino acids. The complete mitogenome sequence of *Penicillium herquei* strain HGN12.1C has been submitted to the GenBank database under accession number CP098395.1.

The dataset enables researchers to investigate fungal genomics, comparative analyses, and secondary metabolite biosynthesis pathways. All data files are publicly accessible and can be downloaded from the respective repositories for further analysis and validation.

## Materials and methods

### Fungal strain culture and isolation

The endophytic fungal strain was isolated from *D. difformis* collected in Ha Giang Province, Vietnam, and was preserved at −20°C for long-term storage at the Vietnam Academy of Science and Technology (VAST)—Culture Collection of Microorganisms (VCCM code: VCCM44283). For experimental procedures, the fungus was subcultured and maintained at 25°C. The isolation and cultivation were carried out according to the method previously described by Tran et al. (2022).

### DNA extraction and genome sequencing

In this study, fungal genomic DNA was extracted using the E.Z.N.A.^®^ Fungal DNA Mini Kit (OMEGA, USA) following the manufacturer’s instructions. The quantity and quality of the extracted DNA were assessed using a Qubit™ dsDNA HS Assay Kit (on a Qubit 3.0 Fluorometer) and by running 0.8% agarose gel electrophoresis, respectively. Library preparation was performed with the SMRTbell Express Template Prep Kit 2.0 (PacBio), followed by polymerase attachment and purification using the Sequel Binding and Internal Ctrl Kit 3.0 (PacBio). Finally, the fungal genome was sequenced using the PacBio Sequel platform with the Sequel SMRT Cell 1M v3 Tray chip and the Sequel Sequencing Kit 3.0 (PacBio).

### Preprocessing and genome assembly

Read quality control and *de novo* genome assembly were performed using FastQC (http://www.bioinformatics.babraham.ac.uk/projects/fastqc/) and the Hierarchical Genome-Assembly Process (HGAP) version 4 (Chin et al., 2013). Genome completeness was evaluated using BUSCO version 5.8.0 (Simão et al., 2015) based on 1,312 single-copy ortholog groups from the fungi_odb10 database, while assembly parameters were assessed using QUAST version 5.3.0 (Gurevich et al., 2013). Repeat sequences in the fungal genome were detected using RepeatMasker version 4.2.1 (https://www.repeatmasker.org/) (Tarailo-Graovac and Chen, 2009).

### Genome annotation and functional analyses

Genome annotation and gene prediction were performed using the Funannotate pipeline version 1.8.17 (Palmer and Stajich, 2020), which integrates multiple gene predictors, including AUGUSTUS version 3.5.0 (Stanke and Morgenstern, 2005), GlimmerHMM version 3.0.4 (Majoros et al., 2004), and the semi-HMM-based Nucleic Acid Parser (SNAP version 2.68.5+ubuntu24.04.1) (Korf, 2004). Within this pipeline, AUGUSTUS was trained using BUSCO genes, with the *Aspergillus nidulans* species model used as a closely related reference to predict gene structures with high accuracy. Additionally, AUGUSTUS gene prediction models were refined using high-quality protein sequence datasets from two closely related *Penicillium* species, *Penicillium chrysogenum* Wisconsin 54-1255 (GCF_000226395.1) and *Penicillium rubens* Wisconsin 54-1255 (GCA_000226395.1), which served as training references to enhance the accuracy of protein-coding gene prediction and annotation specific to the *Penicillium* genus. GlimmerHMM was employed to identify genes with simple exon–intron structures and to detect short genes, while SNAP was used to predict genes with complex structures and alternative splicing patterns, trained using the BUSCO Dikarya lineage dataset. DIAMOND version 2.1.11 was used to filter low-quality gene models, and Evidence Modeler version 2.1.0 (EVM) (Haas et al., 2008) was applied to remove redundant genes and integrate predictions from the three gene predictors into a comprehensive genome annotation. Final gene predictions were based on consensus results to maximize accuracy and completeness. Transfer RNA and ribosomal RNA genes were annotated using tRNAscan-SE version 2.0.12 (https://github.com/UCSC-LoweLab/tRNAscan-SE) and Barrnap version 0.9 (https://github.com/tseemann/barrnap), respectively.

The mitochondrial genome assembly was annotated locally using the MITOS2 pipeline (Donath et al., 2019) within a Conda environment. To ensure high-specificity annotation, analyses were conducted using the RefSeq 89f reference database (fungi-specific) and the Mold, Protozoan, and Coelenterate Mitochondrial Code (NCBI Translation Table 4). Annotation outputs were manually curated to ensure correct nomenclature for ribosomal RNAs (rns and rnl) and to resolve gene fragmentation boundaries. The circular physical map of the mitochondrial genome was generated using OrganellarGenomeDRAW (OGDRAW) (Greiner et al., 2019).

The complete genome of *Penicillium herquei* HGN12.1C was visualized as a Circos plot using the pyCirclize package. (https://moshi4.github.io/pyCirclize/).

## Data Availability

The original contributions presented in the study are publicly available. This data can be found here: NCBI BioProject PRJNA837748, BioSample SAMN54322392.

## Supplementary material

Supplementary Table 1 Gene content and organization of the *Penicillium herquei* HGN12.1C mitochondrial genome

Supplementary Figure 1 Circular physical map of the mitochondrial genome of *Penicillium herquei* strain HGN12.1C. The map shows the distribution of 44 genes, including protein-coding genes (PCGs), ribosomal RNAs (rRNAs), and transfer RNAs (tRNAs). The inner ring represents the GC content graph.
