# Supplementary figures and images for: Complete genome of Penicillium herquei HGN12.1C isolated from Dysosma difformis in Vietnam
Source: Front Fungal Biol. 2026 Feb 2;7:1695025. doi: 10.3389/ffunb.2026.1695025 (PMC12907825; doi:10.3389/ffunb.2026.1695025)

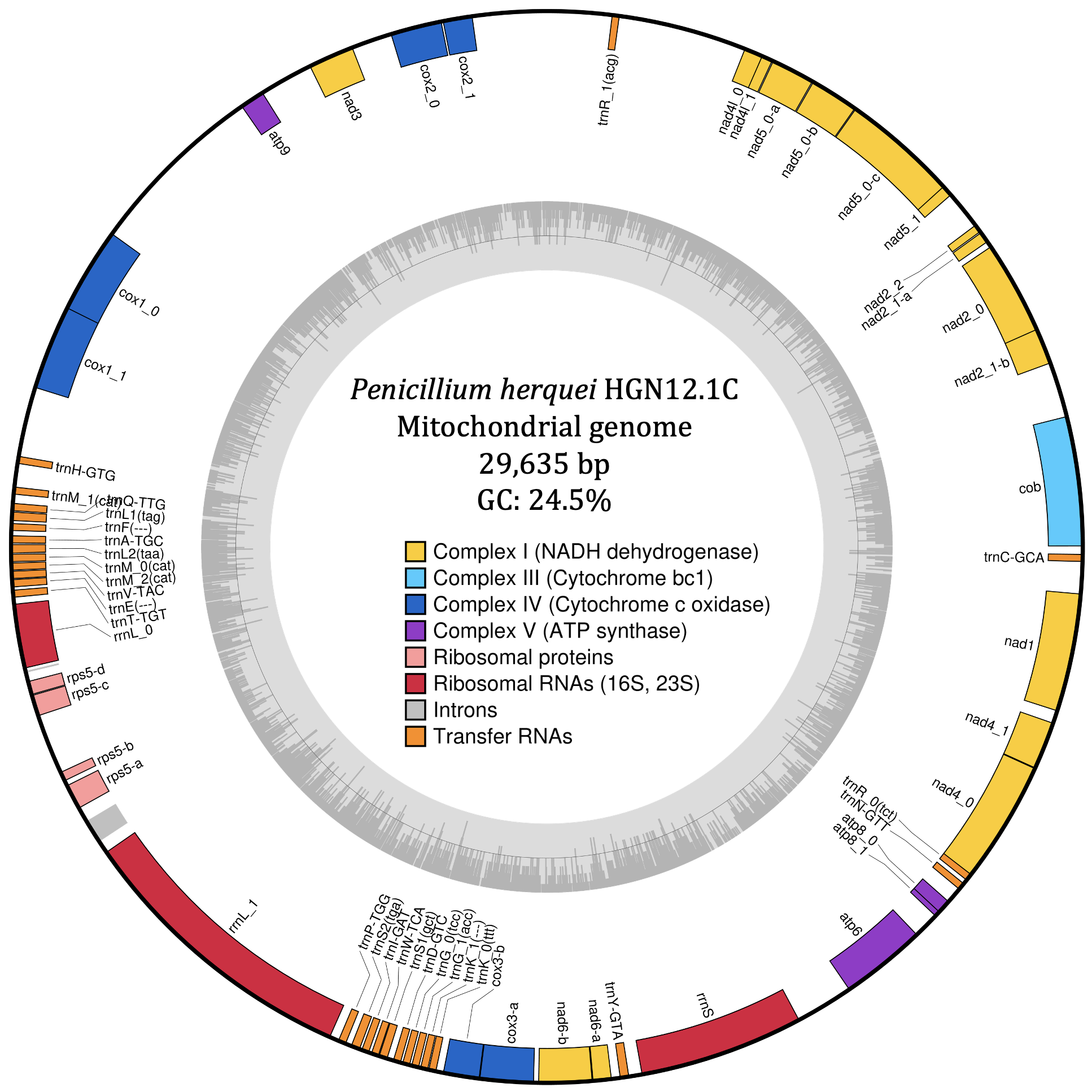

Supplement: Supplementary Table 1 — Gene content and organization of the Penicillium herquei HGN12.1C mitochondrial genome [file SupplementaryFile1.zip › Supplementary material/Figure S1.png]
