# Supplementary material for: Complete genome of Penicillium herquei HGN12.1C isolated from Dysosma difformis in Vietnam
Source: Front Fungal Biol. 2026 Feb 2;7:1695025. doi: 10.3389/ffunb.2026.1695025 (PMC12907825; doi:10.3389/ffunb.2026.1695025)
Supplement: Supplementary Table 1 — Gene content and organization of the Penicillium herquei HGN12.1C mitochondrial genome [file SupplementaryFile1.zip › Supplementary material/Table S1. Gene content and organization of the Penicillium herquei HGN12.1C mitochondrial genome.doc]

Table S1. Gene content and organization of the *Penicillium herquei* HGN12.1C mitochondrial genome

| **Gene Category** | **Gene Name** | Number of Gene copies |
| --- | --- | --- |
| **Respiratory Complex I** (NADH dehydrogenase) | *nad1*, *nad2*, *nad3*, *nad4,* *nad4L*, *nad5*, *nad6* | 7 |
| **Respiratory Complex III** (Cytochrome bc1) | *cob* | 1 |
| **Respiratory Complex IV** (Cytochrome C oxidase) | *cox1*, *cox2,* *cox3* | 3 |
| **Respiratory Complex V** (ATP synthase) | *atp6*, atp8, *atp9* | 3 |
| **Ribosomal Protein** | rps5 | 1 |
| **Ribosomal RNA** (rRNA) | *rns* (16S), *rnl* (23S) | 2 |
| **Transfer RNA** (tRNA) | 27 tRNAs | 27 |
| **Total Genes** | **15 PCGs + 2 rRNAs + 27 tRNAs** | **44** |
